# The Impact of COVID-19 on Sport in Twitter: A Quantitative and Qualitative Content Analysis

**DOI:** 10.3390/ijerph18094554

**Published:** 2021-04-25

**Authors:** Luis-Millán González, José Devís-Devís, Maite Pellicer-Chenoll, Miquel Pans, Alberto Pardo-Ibañez, Xavier García-Massó, Fernanda Peset, Fernanda Garzón-Farinós, Víctor Pérez-Samaniego

**Affiliations:** 1 Department of Physical Education and Sport, University of Valencia, 46010 Valencia, Spain; luis.m.gonzalez@uv.es (L.-M.G.); m.teresa.pellicer@uv.es (M.P.-C.); miquel.pans@uv.es (M.P.); alberto.pardo@uv.es (A.P.-I.); victor.m.perez@uv.es (V.P.-S.); 2 Departament de Didàctica de l’Expressió Musical, Plàstica i Corporal, University of Valencia, 46022 Valencia, Spain; xavier.garcia@uv.es; 3 Instituto Universitario de Matemática Pura y Aplicada, Universitat Politècnica de València, Camino de Vera s/n, 46022 Valencia, Spain; mpesetm@upvnet.upv.es; 4 Departamento de Bioestadística, Epidemiología y Salud Pública, Facultad de Medicina, Universidad Católica de Valencia San Vicente Mártir, Quevedo 2, 46001 Valencia, Spain; fernanda.garzon@ucv.es

**Keywords:** COVID-19, sport, Twitter, text mining, mixed methods, latent Dirichlet allocation

## Abstract

The spread of the SARS-CoV-2 virus has transformed many aspects of people’s daily life, including sports. Social networks have been flooded on these issues. The present study aims to analyze the tweets produced relating to sports and COVID-19. From the end of January to the beginning of May 2020, over 4,000,000 tweets on this subject were downloaded through the Twitter search API. Once the duplicates, replicas, and retweets were removed, 119,253 original tweets were analyzed. A quantitative–qualitative content analysis was used to study the selected tweets. Posts dynamics regarding sport and exercise evolved according to the COVID-19 pandemic and subsequent lockdown, shifting from considering sport as a healthy bastion to an activity exposed to disease like any other. Most media professional sporting events received great attention on Twitter, while grassroots and women’s sport were relegated to a residual role. The analysis of the 30 topics identified focused on the social, sporting, economic and health impact of the pandemic on the sport. Sporting cancellations, leisure time and socialization disruptions, club bankruptcies, sports training and athletes’ uncertain career development were the main concerns. Although general health measures appeared in the tweets analyzed, those addressed to sports practice were relatively scarce. Finally, this study shows the importance of Twitter as a means of conveying social attitudes towards sports and COVID-19 and its potential to generate alternative responses in future stages of the pandemic.

## 1. Introduction

In December 2019, the first cases of pneumonia of unknown origin were identified in Wuhan, China [1]. In the weeks that followed, a new type of coronavirus called SARS-CoV-2 was found to be the main cause of this disease, named COVID-19. Following that, the virus reached Korea and Japan and later spread rapidly to countries on five continents. The World Health Organization (WHO) warned the world of this emergency on 30 January 2020 and declared it a global pandemic on 11 March 2020.

The rapid global spread is evidenced by the increase in cases from 28 January to 30 April 2020 world over. The measures taken to control the situation are unprecedented [2]. The COVID-19 pandemic has challenged the health systems of many countries and has forced health authorities to disseminate information specifically addressed to citizens [3].

Traditional communication channels (e.g., TV, radio, and newspapers) have played a prominent role in disseminating information and developing attitudes during previous pandemics [4]. However, since 2004, the emergence of Internet applications on which users can generate and share content has radically changed people’s approaches towards getting and sharing information [5]. In particular, social networks give voice to all social actors: individuals, companies, and institutions.

The scientific community has studied these new information channels, specifically Twitter, in the context of the most recent health emergencies, such as influenza A (H1N1) [6], Middle East respiratory syndrome (MERS) [7], Ebola [8], and Zika [9]. Since the outbreak of COVID-19, several studies describing the phenomenon of communication through Twitter at different stages during the pandemic have also been published [10,11,12,13,14,15,16]. Twitter and the users involved in the production and dissemination of information on the platform serve as a showcase for researchers to evaluate how generated information is produced, disseminated, and interpreted [17]. As a worldwide social network, it also allows researchers to predict and monitor people’s health behaviors and even change their attitudes or feelings [18,19]. From an epidemiological point of view, this surveillance can have beneficial effects on the control of many people and may even save thousands of lives.

Despite many original articles and reviews published on Twitter and pandemics, the problem we faced while analyzing the information generated is of an overwhelming magnitude, not only because of the number of tweets produced but also because of the variety of topics addressed [20]. This way, when a health emergency occurs, social networks multiply their activity and cover all areas of daily life [21]. For this reason, researchers often deal with small parts of the problem to be able to address it correctly. Obviously, at a theoretical level, any approach provides valuable information on the phenomenon being studied, but because of the urgency in obtaining rapid results that allow us to make decisions soon (i.e., a new outbreak), it becomes a priority to focus on issues that are most representative for the population at the material time [22]. People in an emergency tend to consult familiar sources of information both at the personal (i.e., family and friends) and institutional levels [23].

Nowadays, sport is a key element in contemporary popular culture and a reference for many people’s believes, feelings and behaviors [24]. It is a meaningful phenomenon in negotiating community identity and values [25]. As sociologists have indicated, sport is not only a regulated physical activity but also a cultural, economic, and political phenomenon of enormous relevance to society. Through different theoretical lenses, sociologists have considered sport a social practice, an inspiration for society, religion and opium for people, a business, a quest for excitement, and even a complex global network [26,27,28,29].

Sport is also a ubiquitous phenomenon. Sports news in any media remains universally unavoidable and informs the minds and hearts of people to a great extent the world over [30]. Sports media attracts billion of viewers, who often consume its content daily. Therefore, sport plays a leading role in creating and amplifying the discourses associated with different phenomena and holds great power as a generator of opinions and attitudes. For this reason, analyzing the information on COVID-19 related to sports on Twitter can be useful in identifying how both phenomena circulate concurrently and how sports news can be useful in disseminating health behaviors concerning the pandemic.

Against this backdrop, this study aims to analyze the tweets produced relating to sports and COVID-19. In particular, this paper presents two complementary objectives: (a) to examine how COVID-19 appeared and evolved in the sports tweets via quantitative analysis; and (b) to explore user perceptions contained in the themes inductively and deductively obtained via qualitative analysis. It focuses on sport because it is a remarkable and universal phenomenon that matters in popular culture. Sport is present in many people’s everyday lives, though often is forgotten in academic studies that provide understanding to cultural and social knowledge of health-related issues. Namely, this paper responds to the following research questions: Which are the most frequent words, sources and the main topics of the tweets related to COVID-19 and sport? Which themes provide a narrative description and understanding of COVID-19 and sport on Twitter?

## 2. Materials and Methods

The methodology used in this paper is in line with the necessity spotted by Tinati et al. [31], who highlight the convenience to add meaning to big data obtained from Twitter, combining quantitative and qualitative methodologies to complement the technical capabilities of computer science with in-depth qualitative research methods. This necessity is sustained in Blok and Pedersen’s [32] complementarity idea, which refers to how these different but also essential methodologies can inform each other. In particular, the methodology used in the present study allows, through quantitative algorithms, to zoom the macrostructure of the data, while qualitative content analysis helps to delve into the meaning of tweets at a microlevel. In doing so, the qualitative approach to data allows further development of interpretative steps, while the advantages of quantitative analysis remain preserved [33].

### 2.1. Data Retrieval and Preprocessing

To build our database, a script in the MATLAB (R2019b, Mathworks Inc., Natick, MA, USA) environment was written to perform queries through the Twitter search API. First, only two keywords were used: “coronavirus” and “sport”. The term “COVID 19” was included on 11 February when the WHO recommended using the term as the name of the disease and called for its dissociation from any geographical or animal origin or from other types of coronavirus [34]. These three terms were combined in a single search: tweet_query = “sport (coronavirus OR COVID-19 OR COVID19 OR COVID)”. Please note that “AND” logic is specified in the API with a space between clauses.

Since late January 2020, daily searches were carried out systematically using these terms, collecting all those tweets published on the subject using the ‘recent’ search option. This option in the Twitter API allows the recovery of the newest tweets published in the last seven days. The free version of the API (the one we used) does not give access to all published material, but only to a small part (around 1%) of all material published on the same day and seven days before the search. The only restriction that was applied was that the tweets had to be written in English.

The data were stored in JSON format to extract fields of interest for our study later. The files contained information on each recovered tweet and the user who had produced or retweeted it. These files contained over 150 attributes, of which we used only 11 for this work. This is described further in the analysis section.

The first formal search for our study was carried out on 28 January 2020, and the last one was carried out on 9 May 2020. Over 4,000,000 tweets were recovered in all before the duplicates, replicas, and retweets were eliminated.

**Figure 1:**
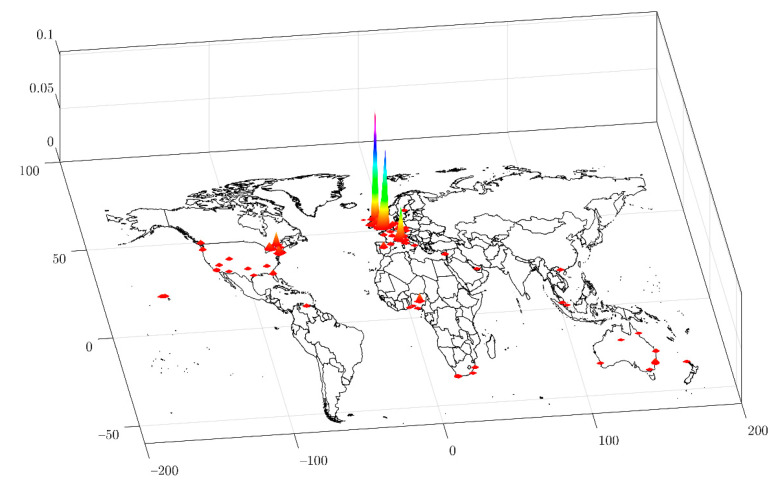
Geolocation of tweets published on COVID-19 and sport. The three−dimensional color bars show the density of tweets (kernel density). Larger size indicates a higher density of tweets in the area. Only those tweets that were geo−tagged are shown (*n* = 4932).

Figure 1 presents the geographical areas where part of the collected tweets are located. Unfortunately, it is unusual for users to geotag their posts (*n* = 4932) [35]. However, most of the tweets collected come from Great Britain and northern Italy.

First, all duplicated tweets were eliminated in the data preprocessing stage using the ID field (unique identifier of the tweet). Tweets that were not original (i.e., retweets) were also discarded. To do this, the JSON retweeted_status field was used. Furthermore, those tweets that began with the expression “RT @” were deleted.

The standard recommendations used in similar studies [36] were followed to prepare the text of the tweets for further analysis. The documents were reduced to tokens (through tokenization), and different actions were carried out in the following order:

- All hyperlinks (“http://url”), hashtags (“# hashtag”), emojis, and username links (“@username”) that appeared in the tweets were removed;
- Punctuation marks and special characters were removed;
- Words were converted to lowercase;
- Words that could add noise to the text and that did not add content to the tweets (e.g., “a”, “and”, and “to”) were removed, using the stopword list that MATLAB’s text analytics toolbox (version 1.4) has by default;
- The words were standardized through a lemmatization process, by which a morphological analysis was carried out to reduce them to their roots. This process uses a predefined dictionary. To improve the process, part-of-speech details were added to indicate whether the word was a noun, verb, adjective, etc.
- Words with under 2 or over 20 characters and whose frequency in the corpus of documents were less than 2 were also deleted.

The resulting tokens were used to form a bag of words (unigrams) and two bags of grams (bigrams and trigrams). An n-gram is a substring of n elements of a given sequence of words. The original documents (raw data) and the associated fields were also stored for qualitative analysis. All other fields containing text (e.g., username, entities mentioned, and places) were not preprocessed in any way.

Finally, although duplicates were located through their unique identifier (i.e., ID), many tweets were published with slight variations from others. For this reason, once the documents were cleaned up, tweets that presented small variations of the original in their text were also removed. After performing all the preprocessing (steps i to vi), the resulting token chains were compared, and those that did not present variations between them were eliminated. In this way, those duplicate tweets that only had punctuation, hyperlinks or morphological variations in some words were eliminated. This was done because this study aims to highlight the original messages produced during the pandemic and not the number of messages sent from one place to another.

The IDs of the tweets used in our analysis can be retrieved from Data S1_id. To comply with Twitter’s terms of service, we are only releasing the Tweet IDs of the collected tweets publicly for non-commercial research use. Users who want to reuse the IDs will be able to recover the original data using some of the existing software in the market (e.g., hydrator). Since the data recovered is public, and many users can be unaware of the relevance or the ulterior use of them, we followed the ethical recommendations proposed by Williams et al. [37]. In particular, no comments on vulnerable populations (e.g., discrimination based on gender, race, or religion) and no mention of individual users who wrote the tweets were mentioned. Only accounts that belonged to public persons or organizations were included in the text without associating them with the content they published.

### 2.2. Quantitative Analysis

The analysis of the tweets began with a description of the database using preprocessed documents and the creation date. The number of tweets per day, the most active accounts, and the tweets that received the most retweets were counted. A frequency analysis of the main n-grams was also carried out. The frequency values of the main unigrams, bigrams and trigrams were represented through word clouds.

To establish the growth and decrease in the most cited words over the study period, the words were represented through a heatmap. All frequency values were normalized using a z-score. Apart from the 20 most frequently used words, some that were closely related to health and hygiene aspects linked to the pandemic were also monitored. This way, through an analysis of the recommendations given by the WHO on its website [38], the following six words were selected to represent the main ways of preventing infection: “hand”, “mask”, “distance”, “clean”, “home” and “lockdown”.

While writing their tweets, authors often pointed to other users. This is often done by including hyperlinks (e.g., hashtags, URLs, or user mentions) from other users or sources of information. In the preprocessing stage, these hyperlinks were removed. However, the mentions were stored in the user_mentions field. With this field, a directed network was built, where the nodes, users, and arcs showed the number of times, a user pointed to another. To describe this net, the following centrality values were calculated using standard functions implemented in MATLAB: pagerank, authority rank, in-degree, weight in-degree, and in-closeness centrality [39].

To establish the topics that emerged from our corpus (i.e., collection of tweets), we applied a latent Dirichlet allocation (LDA) model [40], which assumes the existence of a fixed number of latent topics that appear across multiple documents (in our case, each of the downloaded tweets). Each document is characterized by a mixture of topics, and each topic is characterized by a discrete probability distribution over words; that is, the probability that a specific word is present in a text document depends on the presence of a latent topic [41].

In our study, the LDA model had a double function: it extracted the main topics that users were interested in during the study period from the corpus and served as a method for selecting tweets related to certain topics of interest.

For the analysis, we used the “filtlda.m” function, implemented in MATLAB’s text analytics toolbox (version 1.4). We used the bag of words (unigram) that was previously preprocessed. To ensure that the words used in the search, namely “coronavirus”, “covid-19”, and “sport”, did not distort our results, we eliminated them. This decision was made because our main intention was to analyze the topics that accompanied these words and not these words themselves. Once the bag of words was ready, the first step was to ensure good cohesion in the resulting topics by establishing the number of topics needed. To ensure the goodness of fit of the LDA model, perplexity was calculated previously. It indicates how well the model describes a set of documents. A lower perplexity suggests a better fit. Adjustments were made for 10, 20, 30, 40, and 50 topics. With our data, the number of topics that yielded a lower perplexity value was 30, using a total of 29 iterations. In addition, coherence (intrinsic UMass [42]) was calculated for our data using the 10-top words for each topic. As can be seen in Table 1, the 30-topic solution is the one that shows the highest coherence value. Once the number of topics was established, an LDA model based on a Gibbs sampling algorithm was implemented [43]. All figures and the analyses described in this paper were performed using MATLAB programming environment (R2019b, MathWorks Inc., Natick, MA, USA).

### 2.3. Qualitative Analysis

Following the quantitative analysis conducted around the words and their occurrence, a new complementary categorization was necessary to identify themes that added a narrative qualitative description and understanding of COVID-19 and sport beyond quantitative data. According to Latour et al. [44], both quantitative and qualitative methodologies produce different ontologies of macro- and micro-narratives, but instead of focusing on the distinctions between them, there is an alternative to focus on what is passed from one to the other. In this study, the process of passing characteristics is shown by the selection of the tweets gathered under the 30 topics of the LDA model that emerged from the quantitative analysis. This selection came from tweets that contained 10 more probable words, and the probability of belonging to these topics was over 0.4. This reduction of tweets pretended to focus on the qualitative analysis of the more representative tweets for each topic, avoiding repetitive messages and non-substantial tweets that moved away from the core content. In doing so, the qualitative analysis allowed us to obtain a detailed idea of what Twitter users were talking about, thus complementing and enriching the previous quantitative analysis.

A qualitative content analysis was developed to group the content of the tweets contained in the 30 topics under a more comprehensive, limited, and contextualized number of themes with an in-depth description. A theme is understood here as a thread of subjective meanings that tie similar pieces of data within a cultural-contextual message of data [45].

A summative qualitative content analysis was performed in this study [46]. Compared with other types of content analysis, summative content analysis flexibly explores word or phrase usage from the text and their underlying meanings without imposing preconceived categories or theoretical perspectives. Unlike thematic analysis, which focuses mainly on the manifest content and an in-depth report of commonalities and differences in the data, summative qualitative content analysis takes into account explicit and latent meanings embedded in the content of a data corpus as well as the rich and abstract nuanced interpretations of these data [45].

The analysis began with sampling, the first phase of the content analysis according to Krippendorff [47], which corresponded with the selective representative tweets from the 30 topics in the LDA model. Coding was followed to explore meanings by assigning codes to emergent ideas by comparing, contrasting, checking co-occurrence, omitting, excluding, including, and drawing parallels, among other inductive strategies. The emphasis was more on the emergence of the ideas (inductively and deductively) than their frequency in the building of categories and subcategories for meaning-making, with themes being the more interpretive and global categories that emerged from the data. These themes came up at the end of the analysis and provided a broader contextual understanding of the specific categories. We progressively shaped the categories and subcategories, which finally offered a contour for the four themes in the qualitative content analysis. This procedure of analysis ends in the four themes (social, sporting, economic and health) to provide understanding of the impact of COVID-19 on sport. 

## 3. Results

### 3.1. Quantitative Results

**Figure 2:**
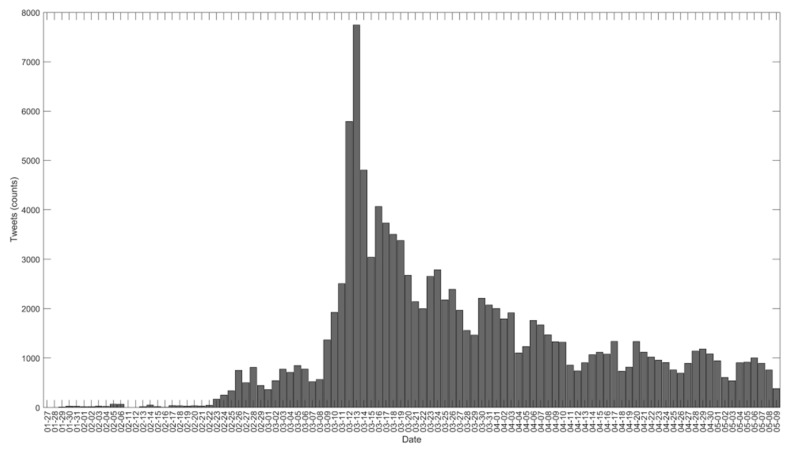
Number of tweets published on sport and COVID-19 during the study period.

From 27 January to 9 May 2020, we recovered a total of 119,253 original tweets on sport and COVID-19. These original tweets were retweeted 345,037 times in this period. Specifically, ≈67% were never retweeted, 32% were retweeted between 1 and 49 times, and only 0.5% obtained more than 50 retweets (see Figure S1_retweets_distribution). The highest traffic of original tweets was seen on 13 March 2020, with a total of 7746 tweets. In Figure 2, we can see the dynamics on the days on which the original tweets were published.

@Mailsport published the largest number of tweets (1073). Two other accounts, @TheSunFootball (923) and @IndyFootball (893), belonging to the two corresponding newspapers, followed next. The main producers of information were corporations, most of which were online newspapers.

A single tweet directly related to the sport that was retweeted most often was published on 29 March 2020 by @MiguelDelaney, Chief Football Writer at an independent journal. This text was accompanied by a generic photograph pertaining to soccer and contained a link to a news item is an independent newspaper. Altogether, it was retweeted approximately 1789 times.

#### 3.1.1. Description of the Words Published in the Tweets (N-Gram) and Their Dynamics throughout the Study Period

The most repeated words in our results were “coronavirus”, “sport”, and “‘covid-19”, which formed part of the search strategy employed. However, one of the objectives of our work was to point out the words associated with these three terms. Table 2 shows the most repeated unigrams, bigrams, and trigrams in our file. Among the unigrams, excluding the words used in the search, the words “league”, “due”, “player”, and “football” were of great importance.

Among the bigrams, “premier league” was most repeated and referred to as a sporting event in England. Finally, among the trigrams, the association between the words “behind closed door” and “coronavirus positive test” stands out. The first referred to the sporting events that were or will be played without an audience, and the second referred to the positive cases found in the tests that were carried out among players. The most commonly used 100 n-grams are listed in Table S1_ngrams.

**Figure 3:**
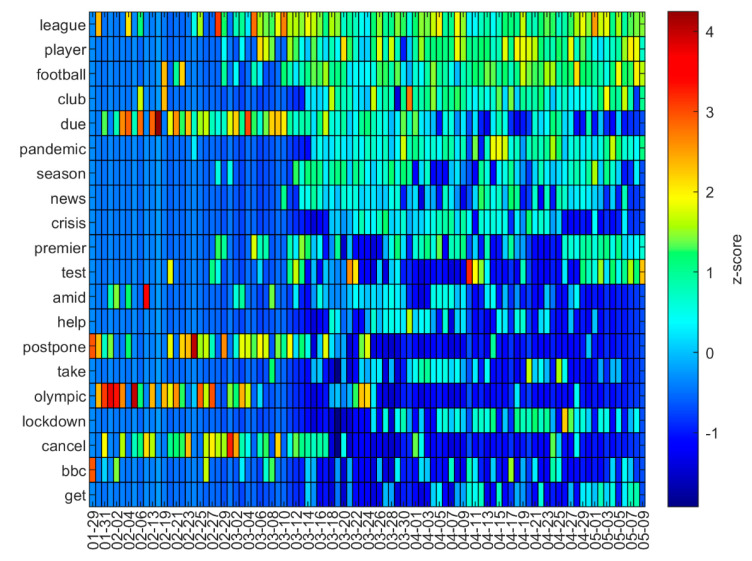
Dynamics of the 20 most repeated words during the study period.

Figure 3 shows the normalized values (z-score) of the 20 most used words. The words “due”, “postpone”, and “olympic” obtained very high values in the early days. From the end of March onwards, the word “test” appears. It bursts out with force at the end of March and in the middle of April, coinciding with the first cases of famous sportsmen testing positive for COVID-19. The word “league” had a similar degree of importance throughout the study period.

**Figure 4:**
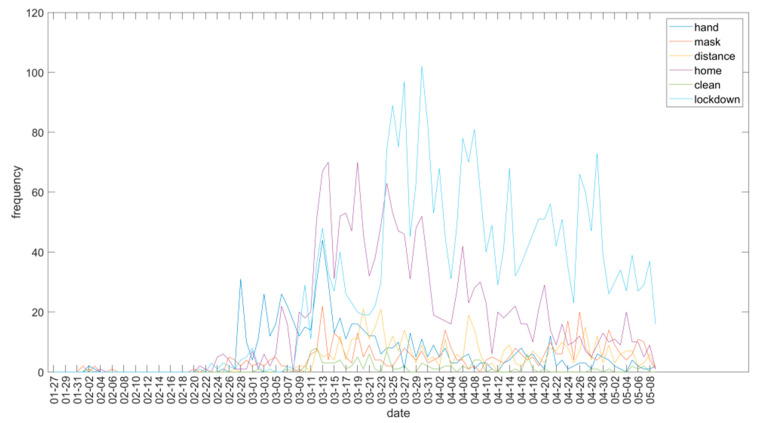
Dynamics of the five terms that are most related to coronavirus prevention measures during the study period.

Besides these 20 words repeated most often, the dynamics of other words pertaining to prevention measures disseminated by the WHO during the study period are also interesting. Figure 4 shows an increase and decrease in the number of times these terms appeared during the study period. The word “hand” bursts in strongly on 2–28, and its prominence is overshadowed from day 3–13 by the word “home”. On the same day, the word “mask” begins to gain a certain degree of prominence.

#### 3.1.2. Network of Mentioned Entities

**Figure 5:**
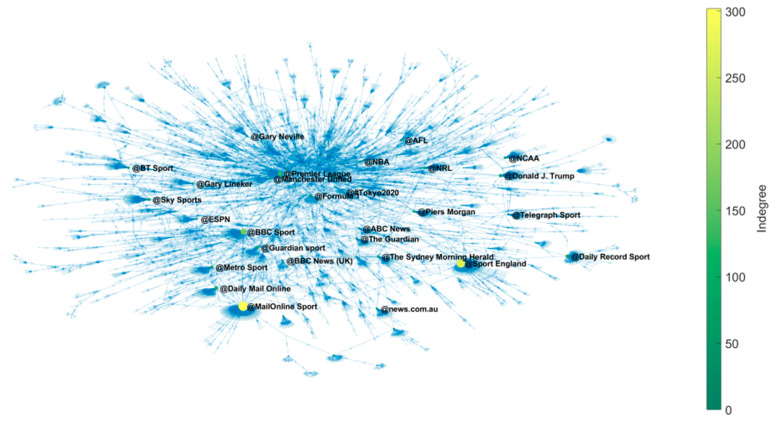
Subnet of mentions with a greater number of components.

Occasionally, users mentioned an entity that supported or contradicted their ideas. These entities are somehow the sources on which the user tweets are based. It is a directed network that has great complexity and comprises an infinity of subnets with a highly variable number of components. However, in this study, we highlight a subnet of 7755 components and more than 12,000 links. As seen in Figure 5, there is a high density of citations, with 29 actors mentioned more than 40 times.

The centrality values of the 20 main actors involved in this subnetwork are shown in Table S2_centrality values. Most entities correspond to digital newspapers, and some to individuals, such as @Donald J. Trump (politician), @Piers Morgan (journalist), or @Gary Neville (former sportsman and coach). There were also some sport event entities and professional sports clubs among these actors, as well.

#### 3.1.3. Main Topics Found in the LDA Model

**Figure 6:**
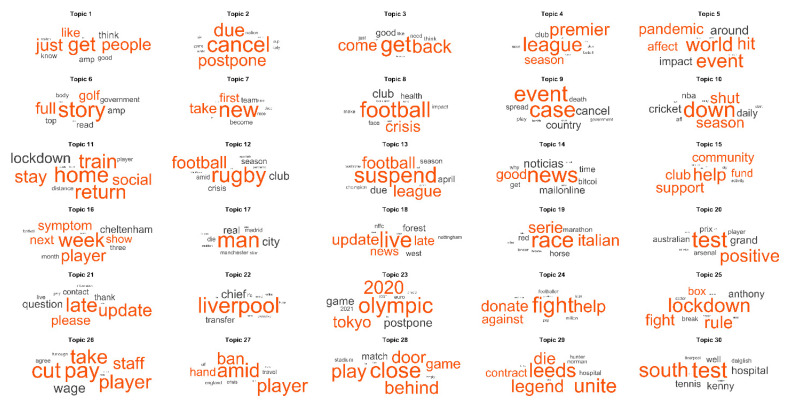
Representative words of the topics found in the LDA model. A larger size represents a higher probability of appearing in the documents associated with each topic. Words with a lower probability appear in a smaller size (for words with a lower probability, see Table S3).

The words were classified into 30 large topic groups using an LDA model. The clusters were ordered from the highest to the lowest probability in the total corpus of documents. Each topic comprised a group of words that had an associated probability of appearing together in different documents. As seen in Figure 6, some words had a high probability of representing the topic in different documents.

A quick glance at these topics gives us a general idea of the main issues that Twitter users, and by extension, the general population, were concerned about in the early months of the pandemic.

The most representative tweets under each of these topics were analyzed qualitatively in the next section of this article.

### 3.2. Qualitative Results

Governments, sport governing bodies, and organizations were forced to make hard decisions since the outbreak of COVID-19. A detailed and updated reconstruction of the decisions is now available, for instance, on BBC’s website (https://www.bbc.com/sport/51605235, accessed on 25 September 2020). See Table S4 (“Themes, categories and example of tweets emerged from qualitative analysis” table).

#### 3.2.1. Social Impact and Significance

Hosting events behind closed doors, postponements, and cancellations triggered complaints and resistance against these decisions. An important group of tweets stated that authorities were “overacting” towards a “mild case of the flu”, and someone even “predicted a backlash from bosses against ‘unfair’ plan to cancel” the sports season. However, tweets also roused criticisms against such complaints and resistance at a time when people were “losing their lives”. Some tweets reflected incensed opinions in favor of people’s health at a critical point in time, such as “Stop being childish”, “Sorry sport, facts don’t care about your feelings”, “I miss sport too, but actual people are dying ffs”, or simply “THERE IS MORE IMPORTANT THINGS IN THE WORLD” than sport.

As the pandemic progressed, tweets suggested that sport consumers had begun to accept the new reality, albeit rather reluctantly. The news of infections, serious health problems, and the death of famous athletes contributed to acknowledging the suspension of events. Boredom was assumed to be a consequence of the lockdown, with people having plenty of time for themselves. As many people recognized, “Life is[was] boring without sports”, “bored now, fucking #coronavirus”, and especially during weekends because they were “just shit”.

According to the tweets, several strategies emerged to fill the gap resulting from the cancellation of sporting events in the lives of sports followers: watching sport hit remakes on TV and YouTube; listening to sports stories on the radio; and watching events that continued to be broadcast from countries that did not have any restrictions. Many people “can just sit in and watch lots of sports”, such as “watching UFCBrasilia”, AFL matches, “the Belarus football league”, and “lost big events” for making time. These tweets showed the power of sport in the lives of many people because they were able to help them cope with the absence of live sport: “E-sports in the time of no sport!”

Some Twitter users reflected on the nature and characteristics of the transcendence of sport itself, as they “wondered what life without sport would be like and now I know”. Others wondered if the sport would look the same after the pandemic, and some even offered a critical view stating that sport should start again, “from bottom-up, not top-down”. Many expressed “how important sport is to everyone”, realizing how much they missed it. As someone stated, “Maybe now people will begin to understand it’s [sic] significance” as “Sport for so many people is an escape from day to day life”. Or, as another one pointed out, “Sport is a huge part of many people’s lives. It’s like a religion to them, so try not to be so dismissive of that”. Sport probably still is, for many people, “the most important of the least important things”.

Certain Twitter users believe that sport has special characteristics that explain its social relevance. For instance, sport allows them to share “things in common with someone at a deeper level” and holds desirable values, such as “to maintain a good, positive attitude” in life. The following tweet explains this idea best: “Nothing unites the world quite like sports, the sportsmanship and competition brings everyone together. The passion that each athlete has for their sport brings positive global interaction”.

#### 3.2.2. Sport Impact

As one tweet stated, “Professional and semi-professional leagues and competitions, as well as grassroots and amateur sports, have been suspended since the middle of March”. This was the first impact of the outbreak of COVID-19 on the sport. It affected the Tokyo Olympic Games and major leagues in five continents. These disruptions had global consequences across the sporting community for many types of sport, especially for athletes, coaches, organizations, and spectators.

Changes in the short- and long-term training of professional and amateur athletes not only had important consequences for their physical and mental wellbeing but also for their immediate careers because of the prevailing sense of uncertainty. As the following tweet shows: “What can athletes and coaches do with the uncertainty that the coronavirus has brought to sports?” In fact, different suggestions appeared for sports professionals, student-athletes, “Parents, and the Sport Community”.

Many tweets expressed concern and worry for the physical performance of athletes “around the world”, as well as for “new ways to stay fit at home during the coronavirus lockdown”. Professionals were “staying at home, some given individual training plans with clubs expecting a mini pre-season before […] start again (whenever that is[was])”. Unfortunately, while referring to young athletes, the cancellation of events because of the pandemic meant that “Some young athletes will never return to their sports even after coronavirus restrictions are lifted”.

Athletes’ broken plans also affected their career development, as seen in the decision-making process that led to the postponement of the Olympic Games. It started with a reaction against it: “Cancelling […] is unthinkable for athletes”. Later, it progressively moved to emphasize the increasing risk of infection and difficulties involved in ensuring proper training, despite the fact that the International Olympic Committee (IOC) urged them to continue preparing (“IOC encourages athletes to continue preparations”, “now athletes like @JohnsonThompson are speaking out about difficulties to maintain training”).

Concerning some sports, particularly football, tweets talked about the immediate career consequences for some professionals because of the difficulties and changes encountered in the transfer procedure. As a tweet pointed out, “The coronavirus crisis will not only resize the transfer market downwards but will also change the perception of figures that we have handled so far”.

#### 3.2.3. Economic Impact

The coronavirus outbreak also provoked a major economic crisis because, without sport, the industry faced major losses as a result of the lack of direct earnings from sports events that were now canceled and indirect earnings from media rights that were suspended as a result. Some spectators demanded reimbursements for “everyone whose made & paid for travel & tickets” and wondered if they had “any chance of getting any money back”. However, several tweets soon showed how the coronavirus outbreak had left “many clubs and organisations struggling financially” because incomes fell abruptly and some “club’s insurers remove[d] pandemic cover[s]”.

To overcome this challenge, professional clubs required sportspersons to take pay cuts (“wage cuts until Premiership returns”, “NRL players told to take 87 percent pay cut to save sport”, “County cricket players to take “maximum” pay reductions”, “Formula 1: Williams drivers take pay cut”, “All Blacks players face 50% wage cut”). They were accepted widely because they allowed non-sporting staff to avoid being fired (“Leeds VOLUNTEER to take wage deferral so non-football staff are paid”, “Barcelona players will take a 70% pay cut […] to ensure non-sporting staff receive full wages”, “Scotland rugby […] agrees 25 percent salary deferral”).

A few tweets on women’s sports referred to the dramatic economic consequences of the pandemic because of the weaker economic nature of their field, unlike men’s sports. “Women’s sport must be safeguarded because coronavirus” and “huge economic impact” “threaten [its] existence”, especially in football, as recent international advancements are under threat.

Economic problems were also observed at amateur and community levels; thus, “Many clubs already rely on retired volunteers, who may be in the ‘at risk’ category and need to stay safe”. These organizations received economic support from sports authorities in different countries to help overcome these challenges. For instance, a total of “£195 million package to help sport and physical activity through coronavirus” and “£20 million Community Emergency Fund available immediately for local club and community organisations” were provided by Sport England.

Finally, economic consequences also affected TV, specialized sports channels, and media houses, which offered reductions in their sports packages (“NENT recently announced it is temporarily reducing the cost of its sports packages” and “Sky announcing that they will allow customers to pause their sports subscription”. With reduced income due to COVID-19, this “would be a very welcome gesture!!”).

#### 3.2.4. Health Impact

In the initial days of the pandemic, tweets tended to minimize the effects of the disease because “people who got the virus had mild symptoms and returned to good health” or attributed the most severe effects of COVID-19 to “older” or “weak” people: “I hate the Coronavirus, not for the fact that it can kill me, but for the fact that it kills weaker people”. In contrast, sports practice was presented as a protection factor, assuming that sport and exercise made people “strong”, “healthy”, and implicitly resistant to COVID-19: “people that dies to the coronavirus are older people that doesn’t really do much sport at all and already has a bad heart”.

Tweets pertaining to safe sport practices and protection measures to be taken in the course of practice exercises were relatively scarce. For instance, in our search, we found no tweets focusing on safety, using masks, or other hygiene measures in sports practice. Only a commercial tweet that referred to a specific mask for cycling was identified: “Buy WEST BIKING KN95 Antiviral Coronavirus Sport Face Mask With Filter Activated Carbon PM 2.5 Anti-Pollution Running Cycling Mask”.

Most sports celebrities’ testimonies were related to general public hygiene measures, such as “stay at home”, “stay safe”, “social distancing”, and “Look out for each other” instead of anything particular to sport practice during the pandemic. However, as the pandemic progressed, some tweets mentioning the importance of the safe and secure practice of sport emerged: “WE WILL GET THROUGH THIS TOGETHER! Golf and sport will bring us all back together, but ONLY when it is safe”. Revival of sports practice is seen as a sign of personal and social renewal and as an important means for personal and social wellness. “We do not know what sport will look like when this is over. We have to hope it looks the same—without forgetting…”. “It’s only when something disappears that you realize quite how much you miss it”. “In this current dark reality, sport doesn’t matter, but it does”.

According to the last tweet, sport and physical activity can matter for health issues pertaining to COVID-19, particularly as a strategy for overcoming stress due to lockdown. As confinement and sporting cancellations spread, tweets referring to becoming active rose both in number and significance. Governmental and private sports organizations as well as individuals and media wrote many messages promoting physical activity and sport among the population and suggested links that people could use to join exercise programs online (“staying active during #coronavirus”, “Great ways to stay active”, “Running is the perfect sport for a pandemic”, “Let us just try & do 10 min × 2 times home exercise”, “‘Join The Movement’ campaign”).

## 4. Discussion

### 4.1. Principal Findings

The COVID-19 pandemic has affected the daily lives of people the world over, as seen in the tweets analyzed in this study. Our results show that since late January and early February, the number of tweets published on COVID-19 and sport began to increase until a peak was reached in mid-March. Singh et al. [48] examined this behavior in a preliminary study. This increase coincided with the days before and after Italy, and Spain took harsh measures to contain the spread of the disease and canceled sporting events to this end [49]. Specifically, the cancellation of some rugby matches in the Six Nations tournament may have triggered this reaction because of the international character of the event (i.e., it affects English and Latin speaking countries at the same time). As shown in Figure 1, the geolocation of tweets (grouped mainly in Northern Europe and Italy) reinforces this idea. In fact, in a study before ours, the geographical distribution of tweets published in Europe, very similar to the one we have reported, was already observed [35].

Much detected traffic came from online newspapers, which have accounts on Twitter where they show a part of their digital content. This presents a bidirectional phenomenon as Twitter is both a site for the publication of news and a source of information for traditional newspapers [13,50,51,52]. According to Engesser and Humprecht [53], it also indicates that “… countries with high journalistic professionalism and large potential Twitter audiences use Twitter more frequently, while countries with low professionalism and small audiences use Twitter less than their respective counterparts” (p.525). As shown before in our results, this was the case for Great Britain, Italy, and, to a lesser extent, Germany.

The most repeated words in all these tweets were those that generally refers to the type of virus, “coronavirus”, and the disease it causes, “COVID-19”. However, both the name of the virus (i.e., at first, the virus was named 2019-nCoV, and later SARS-CoV-2) and how the press should refer to the disease were areas of controversy in the initial days among health authorities. Several historical precedents show the importance of disassociating the name of certain diseases from their place of origin (e.g., the Spanish flu, whose origin was incorrectly associated with Spain) because it can generate significant stigma on people from and living in the place in question [54]. For this reason, the media avoided using the term “Wuhan” to label the pathology associated with the virus. Our results indicate that at least in sport-related tweets, the city and the pathology were dissociated [55].

From a sporting perspective, the words “league”, “due”, “player”, and “football” were most repeated on Twitter during the period of analysis. European football had a prominent presence in this social network during the study period, although the word “football” was used to refer to different sports in English (i.e., football, American football, rugby football). The word “NFL” (National Football League), which is associated with American football, appeared in the 596th position of the world ranking. From this, we can deduce that the impact of the coronavirus on US events was discrete during the study period. Future work should focus on other periods during the pandemic to establish the impact of COVID-19 on American football, and by extension, on American professional sport.

The word “olympics” also had a prominent place in many tweets. The fact that the pandemic began in Asia, and Japan was one of the first to be affected, fostered the appearance of many tweets on the possible impact on the Tokyo 2020 Olympics at the end of January 2020. The Olympic Games are the largest economic, political, and social sporting event in the world and decisions on this macro-event can affect many other sporting events.

The quantitative results also showed that words related to prevention had a relatively low presence. For instance, the word “mask” hardly appeared in our analysis. Early doubts about its usefulness [56] may have influenced the unpopularity of the word. In contrast, preventive measures associated with hands (i.e., not shaking hands and washing them) were present from the beginning. However, the most remarkable thing is that the frequencies of all these words are very low. As with previous studies conducted during other pandemics [57,58], it seems that words indicating prevention measures are not among the most popular.

Most users mentioned the news from particular newspapers. However, only very few tweets mentioned the URLs of national or supranational institutions that were not related to sport [59]. This suggests that tweets about sport were not related to the official information provided by governmental institutions. This is relevant because the number of people who read only sport-related news was very large. Consequently, we can infer that a high percentage of users did not link their messages to the prevention measures given by official agencies. Although sports newspapers usually include health information, it is usually of low quality and focuses on the injuries of soccer players or doping [60].

The qualitative results indicate that the pandemic has affected many facets, persons, groups, and organizations at different levels in the sport system. Tweets under study in this paper focus especially on the social, sport, economic, and health impacts, as seen in the main themes identified in the analysis.

The tweets studied to prove the powerful role that sport plays in the lives of many people. They realize it when the sport is snatched from their lives, breaking their daily routines abruptly. Many of them realized sport provided the excitement they needed to endure their routinized ordinary lives, as identified by figurational sociologists [27,61]. This was especially observed in the Twitter user resistance to the postponement and cancellation of sporting events and leagues and their strategies to address the boring period without live sport. Playing e-sports and watching sport hits on TV or YouTube became the main substitute while they waited for “real” sport.

Some tweets emphasized how the pandemic had disrupted their main ways of socializing with other people and establishing ties with their family and peers, as Parnell et al. [62] identified. Others referred to the desire to recover positive social and personal outcomes assigned to sports as experienced before the outbreak that, according to Coakley [63], reminded one of the socialization effects taken for granted in Western societies since the emergence of modern sport. These messages continue to show how people value sport as a self-evident good and the inspirational role they assign to it for the entire society, affirming what Delaney [64] identified as a functionalist view of sport that supports the social order.

The cancellation of sporting events and the lockdown to contain the spread of COVID-19 had short- and long-term consequences, especially for athletes and coaches. Twitter referred to the stress on their health and the uncertainty it provoked in their careers. For instance, users referred to the transfer process that was affected at the professional level and the broken expectations that prevailed at the grassroots level because young athletes are in the early stages of their career. This is an important issue that Evans et al. [65] highlighted while referring to the effects of postponements on athlete wellbeing and sense of self, as well as in their career progressions or early retirement. These authors referred to the consequences of physical distancing in the course of training and sporting interactions and how security measures affect “sport for all” because of the risk of infection in some groups (e.g., older adults, pregnant women, and chronically ill people). The question remains as to whether sports will be the same after the pandemic, as some tweets in this study also mentioned.

Our results indicate that the sporting industry and the economic consequences of COVID-19 received important attention on Twitter. Reimbursement of money after the postponement and cancellation of sporting events and the lack of revenues were emphasized in some tweets. A recent study showed how the Big Four League in North America supposed a potential revenue fallout of 6bn dollars if they did not collect any gate receipts until the end of the season [66]. However, many other economic facets are mentioned in our data on the reduction of income from sponsors, broadcast revenues, merchandising, and travel and accommodation, which may produce an extension fallout to the tune of 160bn dollars [67]. This puts sports organizations on the brink of bankruptcy, affecting the salaries of athletes and non-sport staff, the transfers of players, insurance problems and preventing other investments from being realized. All these consequences were emphasized by most organizational position papers and reports [68,69,70]. They were found to affect both professional level and amateur and recreational sport. There are also adverse impacts on the exercise and health industry. Athlete or player wage cuts and salary deferrals were perceived as relevant contributions to overcoming the strong economic crisis in the sports sector. Some tweets and organizations referred to the more severe repercussions on women [71] and on community sport that receives economic support from the governing bodies of other countries.

The sport appeared in many early tweets in the form of an intrinsic factor of protection, a sort of magic shield that protects people from serious health problems. Previous research has revealed that this attitude is typical of male, young, sporting bodies facing illness [72]. It also revealed how sports celebrity testimonies were related to general public hygiene measures, but few of them were focused on the measures that were specifically centered on sports practice. In this sense, it is doubtful that Twitter helped gain awareness of the health consequences of COVID-19 (including protection measures), at least concerning sports practice. It is important to consider that the age profile of Twitter users (38% are young adults aged between 18 and 27 years) coincide with the population that is currently more reluctant to adopt protection measures to keep COVID-19 at bay [73,74]. However, messages on the necessity of staying active also spread through Twitter to compensate for the negative physical and sociopsychological effects of confinement [75,76]. It was also suggested that training regimens and healthy behaviors should be introduced during pandemics as standard habits for health and wellbeing [77].

### 4.2. Limitations and Future Research

The first limitation of our study is directly related to the language used for the search. We chose to collect tweets in English alone. Although English can be considered the lingua franca on Twitter [78], this decision may have underestimated and minimized the opinions of some users from other countries who do not use English regularly. Future research should include other languages and localize the exceptions that may have emerged in other languages. The limitations imposed by Twitter on unpaid searches should also be addressed. Our search did not cover the entire universe encompassed by Twitter, as it only comprised a sample of what was published. It must also be stated that accounts marked as private cannot be retrieved with Twitter API. Another aspect that must be addressed is our decision to analyze only original tweets. Future analyses should focus on replicas and retweets produced, as that may present new results complementing our findings.

Beyond these limitations, future work should focus on athletes and sports institutions with a significant presence on Twitter. Particularly, it would be desirable to track the news published about COVID-19 in the accounts of professional athletes and the sports newspapers with a presence on Twitter.

## 5. Conclusions

This paper presented how COVID-19 has affected most facets of the sport, such as governmental organizations, clubs, athletes, managers, coaches, and fans, as well as the sporting community on the whole. The quantitative dynamic of posts on Twitter concerning sport evolved according to the pandemic and lockdown to contain its spread. However, many of these tweets were created around most media sports events (e.g., football leagues and Olympic Games) generated in the news agencies and newspapers. Our quantitative and qualitative analyses show that Twitter focused mainly on professional sport and mass entertainment events, whereas grassroots and women’s sport was relegated to a residual role despite the adverse impacts of COVID-19 on those areas, the career development of the athletes involved, social advances, and health consequences. No tweets referred to other vulnerable groups, such as immigrants, the homeless, and refugees, and potential groups segregated under virus risk (e.g., old people). This shows how COVID-19 had spurred new forms of economic and cultural inequalities that should be considered if the pandemic continues to spread.

The economic impact has devoted important attention to the messages on Twitter, as has happened in the economy in general, coming to dispute with the preservation of people’s health messages during the initial sports decisions on postponements and cancellations. These disputes and tensions were softened at the time when the athletes, coaches, and young fans were not as invulnerable as Twitter users expected them to be. Messages on sport shifted from considering this social practice as a healthy bastion to an activity exposed to disease like most other activities.

However, the counting of tweets on sport exhibited that neither the media nor Twitter users focused on general hygiene measures. The qualitative analysis showed the resistance among Twitter users in the early stages to adopting health-related measures in mass events. Although sports celebrities participated in prevention campaigns and cancellations contributed towards reducing the spread of COVID-19, it is doubtful whether Twitter helped gain awareness on the health protection measures. This is especially dramatic as sports is a universal practice with a worldwide audience. However, our qualitative analysis showed that people were mainly worried about how to fill the gap left behind in the wake of the cancellation and postponement of sporting events and to address the boredom ensuing as a result, rather than being concerned about protecting their health. Health authorities should take advantage of the opportunities offered by sport for communication through media and social networks to transmit information on health protection and prevention measures in the future. In particular, the enrollment of most media sports persons could play an important role in the dissemination of messages about preventive measures. The practical implications of our study point in this direction. Our results may suggest that Twitter and, specifically, sports-related tweets can be a vehicle for the dissemination of messages on the pandemic, using professional sports celebrities as loudspeakers. The following recent tweets about the position of two world-renowned tennis players (Andy Murray and Rafael Nadal) regarding vaccines are good examples: “Murray supports vaccine in tennis” or Nadal: “Vaccine is the only solution to go through the pandemic. We need to accept it. I know some people have side effects, but the coronavirus effects are worse. I will get vaccinated if I can”. However, despite the great potential support for vaccination, these messages have had little impact on institutional user accounts. Conversely, policymakers, public health and sports professionals, and the media should pay closer attention to the possibilities of sport in Twitter for delivering anti-COVID-19 information. The next Tokyo Olympic Games this summer of 2021 offers a golden opportunity to put this strategy into practice.

Finally, an important question emerged from this study for the near future: Can the interest in exercise and sport observed in Twitter serve as a new impulse for improving people’s quality of life instead of a simple confinement compensation?

## Figures and Tables

**Table 1:** Coherence measures for the choice of the number of topics in the LDA model.

Top Words	Number of Topics				
	10	20	30	40	50
10	–51.645	–51.580	–51.137	–52.367	–54.028

**Table 2:** The unigrams, bigrams, and trigrams that were used most in the tweets recovered.

Uni-Gram	Freq.	Bi-Gram	Freq.	Tri-Gram	Freq.
coronavirus	57,482	premier league	5573	test positive coronavirus	1675
sport	32,232	due coronavirus	4428	behind close door	1637
league	10,346	coronavirus pandemic	3598	bbc sport coronavirus	1185
covid-19	9552	amid coronavirus	3079	premier league club	744
football	7754	coronavirus crisis	2833	amid coronavirus crisis	695
player	7441	test positive	2786	play behind close	635
due	7040	bbc sport	2692	amid coronavirus pandemic	633
season	6164	coronavirus outbreak	2581	due coronavirus pandemic	578
premier	5945	sport coronavirus	2074	cancel due coronavirus	548
club	5860	positive coronavirus	1878	coronavirus premier league	542

## Data Availability

All the data are included in Supplementary Materials.

## Supplementary Materials

The following are available online at https://www.mdpi.com/article/10.3390/ijerph18094554/s1, Data S1_id; Figure S1_retweets_distribution; Table S1_ngrams; Table S2_centrality values; Table S3_top_word_topic_LDA; Table S4_example of tweets emerged.
