# Supplementary material for: The Impact of COVID-19 on Sport in Twitter: A Quantitative and Qualitative Content Analysis
Source: Int J Environ Res Public Health. 2021 Apr 25;18(9):4554. doi: 10.3390/ijerph18094554 (PMC8123335; doi:10.3390/ijerph18094554)
Supplement: Supplementary file 1 [file ijerph-18-04554-s001.zip › Table S4_example of tweets emerged.pdf]

STable. Themes, categories and example of tweets emerged from qualitative analysis

**Theme**	**Categories**	**Examples of tweets**	**Date**
S<br>O<br>C<br>I<br>A<br>L<br><br>I<br>M<br>P<br>A<br>C<br>T<br><br>A<br>N<br>D<br><br>S<br>I<br>G<br>N<br>I<br>F<br>I<br>C<br>A<br>N<br>C<br>E	Complaints and resistances	- Im soory (sic) but people are way too overreacting, people that dies to the coronavirus are older people that dosen't really do much sport at all and already has a bad heart.<br>- Never before, in the history of sport... But, no worries, it's just a mild case of the flu<br>- Well you may as well suspend sport forever then because the flu kills more people than coronavirus.<br>- Even though coronavirus only stays airborne for a few seconds and cannot survive in sunlight for very long. Oh and softball is an outdoor sport. Oh and 80,000 people who got the virus had mild symptoms and returned to good health 10-14 days.<br>- This week: everyone taking it serious as fuck because sport is cancelled.<br>- Obviously people give a fuck about the threat of coronavirus & the loss of lives, but they are entitled to give a fuck about other things too, even if you don't.<br>- BILLY BROWN [League Managers' Association chief] has predicted a backlash from bosses against 'unfair' plans to cancel the Scottish football season and honour current league positions.<br>- By the time #coronavirus is over, I will have forgotten why i like sport	2 March<br>3 March<br>10 March<br>12 March<br>13 March<br>13 March<br>16 March<br>6 April
	Resistance and complaints criticism	- That's what we like to see, keep this so called sport going, sod the Coronavirus, money is more important, not the welfare of the public.<br>- We obviously mustn't upset big business or sport because, well, you know, they are much more important than staying alive.<br>- Sorry to say it but… you watching your favorite sport is NOT more important than people LOSING THEIR LIVES.<br>- The one thing that Coronavirus has done extremelly well is exposing the idiots. Not only those who make sick jokes when people are dying, but also who put their own club interests over the future of the sport.<br>- There are a lot more important things in life than a golf tournament.<br>- Sport is a big part of our lives, but it's really not that important in comparison to keeping people healthy.<br>- Spoiler: if you have a pre-exiting condition but your lungs stop working bc you contracted COVID-19, the pre-existing condition didn't kill you, COVID-19 did. Sorry sport, facts don't care about your feelings	11 March<br>11 March<br>12 March<br>13 March<br>17 March<br>14 April<br>20 April
	Accepting reality and preparing for boring	- Literally the BEST thing to EVER happen to this boring, lame, shitbox sport. Thank god for the #coronavirus<br>- I know sport isn't the most important thing in all this but it truly is surreal<br>- With the #CoronaOutbreak going on, all sport seems to be getting cancelled. Definitely the right thing to do, but god it may be a boring couple weeks!<br>- I'm that bored now, fucking #coronavirus has ruined my life. No sport on for the foreseeable future. Looks like Twitter is gonna have to consume that empty void now<br>- Life is boring without sports…Would you allow yourself to get #Coronavirus if it meant getting every sport back?<br>- I've never really got these 'I'm not really into sport' types of lads...even more so now! What the fuck do they do without sport? It's been 3 days and I can't function<br>- I'm sorry but this Coronavirus needs to fuck off. Weekends without sport are just shit.<br>- The fucken coronavirus is so fucken dumb why the fuck is this even a fucken thing. This ruin me a lot im bored, i dont get to see my friends, i dont even get to play my fucken favorite sport. Fuck this shit bro<br>- Dear #coronavirus give us sport back. Things are getting out of hand	12 March<br>13 March<br>13 March<br>14 March<br>14 March<br>15 March<br>15 March<br>20 March<br>17 April

	Strategies to refill sport gap	- Seems like a good time to invest in @FortniteGame . Seems it may be the only “sport” left you can watch online.<br>- Need a sport to start watching during #coronavirus isolation.. start watching #UFCBrasilia .. these people beat the crap out of each other.. they're not scared of a virus<br>- Well, seeing as we are not really supposed to be going out, I can just sit in and watch loads of sport. Oh........#coronavirus<br>- F1: Max Verstappen and Lando Norris find alternative ways to race as F1 stars turn to eSports: Motorsport has been cancelled entirely due to the coronavirus crisis, but stars from F1, IndyCar and Formula E all found ways of competing at the weekend as… http://dlvr.it/RRz7pV<br>- I’m watching Halladay’s playoff no-hitter on Youtube. Coronavirus can’t stop me from watching my favorite sport, damn you<br>- After the last few days of just being hit with COVID-19 everywhere I look on TV, socials and in my work, sport would be a great distraction. I’m m gonna be playing a lot of video and board games.<br>- Hey Americans, if y’all need a sport to watch, try out the AFL, 7:40pm AEST (Australian time) tonight. Best game in the world! #coronavirus #AFL<br>- Life in the time of #Covid_19 How to amuse yourself at home idea 1 - unearth your old Wii Sport and start playing<br>- To anyone missing @NBA, @NHL and anything else there is hope: The Belarus football league is still playing baby! #football #soccer #COVID—19<br>- My thoughts on esports during COVID19. E-sports in the time of no sport!<br>- Good Sunday, everyone! I woke up to watch #eNASCAR and it's kinda cool! It looks like you're watching a real NASCA<br>- Much like every major sport, @iplaycornhole lost big events (& a big TV window opportunity on ESPN) as the coronavirus pandemic spread. But now it's leaning on a tool that allows players to compete virtually to help maintain cornhole's upwards trajectory<br>- I get that things are serious at the moment but what is the point in a @SkySportsNews channel just now? All they're showing are updates that should be on @SkyNews. Stick to how #coronavirus is affecting sport and leave the news on @BorisJohnson to your Sky News colleagues	12 March<br>14 March<br>15 March<br>16 March<br>16 March<br>18 march<br>19 March<br>22 March<br>29 March<br>29 March<br>29 March<br>6 April<br>7 April
	Sports role in people’s lives	- Sport is a huge part of many peoples lives. It's like a religion to them, so try not to be so dismissive of that.<br>- Sport for so many people is an escape from day to day life, it’s important and maybe now people will begin to understand it’s significance. The impact mentally for people of a blanket ban (which I agree with) will be huge keep an eye on your mates and family #Covid_19<br>- Always wondered what my life would be like without sport and a healthy social life with friends. A harsh way to find out what it's actually like. Hope we're back to normal by Summer. Stay safe folks #COVID19<br>- Coronavirus really makes me think about what I've got in common with my dad... Sport is the ONLY thing, and since a…<br>- It’s not until something like this (Covid-19) happens that you realise how important sport is to everyone. Whether you watch it or listen to it, it’s always there. I feel slightly lost without it.<br>- Coronavirus: 'In this current dark reality, sport doesn't matter but it does'. Its only when something disappears that you realise quite how much you miss it, says BBC Sport’s chief sport writer Tom Fordyce.<br>- Sport is life, in all its beauty and all its heartache. I think the important thing in life is to maintain a good, positive attitude and maintain connection to yourself and to your God, whatever that may be." Amen<br>- Finding out you have things in common with someone on a deeper level than “we both like the same movie or sport” is the most compelling and energizing thing ever. I don’t want quarantine anymore, I was to be with people.	13 March<br>13 March<br>14 March<br>15 March<br>15 March<br>16 March<br>24 March<br>12 April

		- Nothing unites the world quite like sports, the sportsmanship and competition brings everyone together. The passion that each athlete has for their sport brings positive global interaction. Hoping the 2020 Olympics can resume next year despite COVID-19.<br>- Interesting idea, this, though it'll never happen. Start sport from the bottom up. Coronavirus: Sport should start again 'from bottom up not top down'	27 April<br>29 April
Sport impact	Postponements and suspensions	- Coronavirus could have a huge impact on major sporting events this year. @amyyyforbes looks at the effects coronavirus has had, and could potentially have in the sporting world.<br>- Italy on alert as 40 semi-professional games are postponed due to coronavirus fears<br>- Olympic athletes told to 'focus on your sport' ahead of Tokyo 2020 despite coronavirus fears<br>- Six Nations games under threat and five Serie A matches to be played behind closed doors because of CoronaVirus<br>- Inter Milan's chief says serie A season runs risk of not finishing if more football matches are postponed because of coronavirus<br>- Celtic would NOT be guaranteed Premiership title if coronavirus leads to mass cancellations<br>- 'I ain't playing without fans': LeBron James will refuse to play if NBA games go behind closed doors<br>- Ok No esport. No real sport BBC Sport - Coronavirus: Premier League and EFL suspended until 3 April at earliest<br>- "This is just the start of the Coronavirus, in UK. Very little chance there be any sport started again before end of May. Look at situation in China nearly three months on from case."<br>- Nottingham Forest news | The Reds' season has been suspended due to COVID-19 outbreak, with Forest, Leeds United and West Bromwich Albion among a host of clubs striving to be promoted to the Premier League<br>- Leeds fans rage as West Ham chief Brady demands season to be void over coronavirus fear<br>- Coronavirus: Liverpool not winning title 'unjust' says Brighton chief executive Paul Barber<br>- BBC Sport - Coronavirus: English rugby union's Premiership set to suspend season<br>- Team GB athlete calls on IOC to postpone Olympics due to coronavirus<br>- 2020 Tokyo olympics postponed to 2021 amid coronavirus pandemic.<br>- Ilkay Gundogan gets it RT @BBCSport: "You have to be fair as a sportsperson." Ilkay Gundogan says it would only be fair to award the Premier League title to Liverpool if the season is not completed because of coronavirus.<br>- Tokyo Olympics will be cancelled if it cannot be held in the summer of 2021, says president of committee<br>- The coronavirus pandemic and subsequent lockdown has hit sport hard – @jdgmedia takes a look at how different sports in the UK are dealing with the issue and where they're at in terms of restarting.	6 February<br>22 February<br>26 February<br>26 February<br>1 March<br>2 March<br>7 March<br>13 March<br>14 March<br>14 March<br>14 March<br>14 March<br>15 March<br>16 March<br>23 March<br>30 March<br>29 April<br>6 May
	Sport persons and community's wellbeing	- Are you, or is someone you know, feeling isolated by the lack of sport? Feel free to get in touch and keep the conversation flowing during these uncertain times.<br>- Here are some great tips! The COVID-19 Pandemic: Tips for Athletes, Coaches, Parents, and the Sport Community<br>- We're all in this together- check out these new resources from the NCAA on 8 strategies to help you and your student-athletes. [mental health]<br>- I spoke to @baldocktownfc boss @MrFish82 earlier this week and he raised a serious concern. How is the #coronavirus suspension impacting the mental health of non-league footballers?<br>- Rea warns of 'huge problem' in mental health during coronavirus crisis	20 March<br>20 March<br>25 March<br>26 March

		- Our very @GeorgieGoalball sharing fantastic tips for our mental and physical health during these challenging times<br>- Topic #1: Mental Health. If you're struggling, please speak up! We can help. @NCAA provided great tips on how to cope during this time and resources to use if needed. Please click on the link and read!<br>- "Scrap it and start again. Start it again yeah. It's gotta be you know. If we can't carry it on it's got to be void. "Fans are so important. You realise it even more [now]. I think the sport is for fans really," Luke Shaw says<br>- The Sport Wellbeing Hub is a valuable online resource that we encourage everyone to use across the sporting community, those involved in sport, at all levels and all abilities. Everyone.<br>- Sport NI, in partnership with the PHA have launched The Sport Well-being Hub in response to the COVID-19 challenge. Some fantastic information and inspirational messages from our sporting heroes<br>- Anyone interested in football, mental health and the unprecedented impact of COVID-19 on elite sport organisations must listen to this! The perfect soundtrack to your daily walk<br>- "The majority of players are scared because they have children and families." Manchester City striker Sergio Aguero says players are fearful over a potential Premier League return amid the coronavirus pandemic.<br>- "Everything's coming back int o the same thing – why should anyone starve in this world, right? Why?" - an interesting chat with Grimsby boss Ian Holloway about the health of lower leagues, the need for change across society & hanging on during lockdown.	27 March<br>29 March<br>30 March<br>6 April<br>24 April<br>24 April<br>1 May<br>1 May<br>4 May
	Athletes' physical performance	- Great initiative from our partner NGO @AMATKDGYM #SportsDiplomacy sharing #parataekwondo exercise videos to help keep their extraordinary students fit.<br>- Premier League in lockdown like the rest of us. Players staying at home, some given individual training plans with clubs expecting a mini pre-season before we start again (whenever that is)<br>- Ben Stokes on challenges of maintaining training regime during coronavirus lockdown<br>- Liverpool coach tells Alisson what he must do during coronavirus lockdown<br>- Around the world, athletes are working on new ways to stay fit at home during the coronavirus lockdown<br>- Klopp opens up on how Liverpool stars are dealing with coronavirus lockdown #LFC	21March<br>24March<br>29March<br>21 April<br>24 April<br>5 May
	Career disruptions	- The @NCAA should grant every spring sport athlete another year of eligibility. It isn't their fault their season got canceled. They are all victims of unfortunate circumstances with the Coronavirus. They shouldn't be punished due to things beyond their control<br>- Summer transfer window and contracts to change amid coronavirus epidemic as FIFA set up plans<br>- #askCarli with the coronavirus shutting down spring sports-what advice would have for all spring sport athletes while they wait for their chance to play?<br>- Coronavirus could force new transfer window as Liverpool told to brace for change.<br>- Barça will have to sign differently: The coronavirus crisis will not only resize the transfer market downward but it will also change the perception of the figures that we have handled so far.<br>- Sport Contact USA helps soccer players find college soccer programs! Even with Coronavirus, college soccer recruiting is still happening. Electronically marketing with professional highlight video, proper emails,...<br>- "It feels like all the hard work - I don't want to say it's for nothing, but I want to compete" For Many #Athletes, #Coronavirus Means the End of College Careers<br>- Despite the disruption caused by the COVID-19 to sport participation worldwide, two local young wrestlers are serving as excellent	12 March<br>18 March<br>20 March<br>22 March<br>27March<br>28March<br>28 March

		ambassadors for wrestling in South Africa where they are currently studying in America.<br>- Premier League 'big six' stars' transfer values plummet due to coronavirus<br>- Some young athletes will never return to their sports even after coronavirus restrictions are lifted, says a South Australia academic.<br>- COVID-19: Kerala's blind cricketers struggle for a ray of hope. Lockdown robs many of them, who made a living from daily earnings, of livelihood	8 April<br>12 April<br>7 May<br>7 May
Economic impact	Professional level	- Wonder if the @Federugby @EnglandRugby or @SixNationsRugby are going to reimburse everyone whose made & paid for travel & tickets<br>- Seems as tho we can't watch any sport #football #golf #F1 is there any chance of getting any money back @SkyUK<br>- Coronavirus outbreak could see Prem stars out of contract sign 'weekly deals'<br>- No games, big losses: Money crisis faces US Olympic sports<br>- Really good insight into Morecambe FC. A lot of really decent people involved with the Shrimps. No multi-million pound deals there, just honest graft...<br>- USA Rugby to file for bankruptcy due to coronavirus USA Rugby will file for Chapter 11 bankruptcy due to "insurmountable financial constraints" that were accelerated by the coronavirus pandemic, the governing body said on Monday.<br>- I fear for the future of sport after the COVID-19. Business will feel the pressure post COVID-19 and might/will be forced to cut budgets. Guess where they mostly look at when doing budget cuts?<br>- New Zealand Football asks Fifa for financial assistance during Covid-19 pandemic<br>- "We have taken the necessary steps to protect all our employees and the interests of the football club." FC Halifax Town confirm they have placed all their staff, except one, on furlough leave due to the coronavirus pandemic.<br>- 'There will be a huge financial hit': cricket clubs count cost of lockdown<br>- Wow. Another sport down because of COVID-19. You just feel for all of those employees that are out of a job right now. We just hope that this doesn't mean the entire league is done and that they are saving money for future years. The #XFL was looking good.<br>- The Tangerines' boss spoke on the possible financial implications of the domestic football suspension due to coronavirus, and what could possibly come next.<br>- @Howden_UK experts look into the impact of the Covid-19 pandemic on sports event cancellation insurance, and summarise some of the most frequently asked questions posed by rights holders and event organisers<br>- It is estimated that US pro sports would lose $5.5 billion from the shutdown while college sports would lose $3.9 billion and youth sport tourism would fall by $2.4<br>- Ross Brawn has revealed Formula 1 is planning to reduce the amount teams can spend under the new budget cap by $30m from the original figure of $175m, as the sport looks to cut costs in the wake of the coronavirus pandemic.<br>- Cricket: Coronavirus could cost ECB £380m - Tom Harrison	4 March<br>13 March<br>16 March<br>28 March<br>29 March<br>31 March<br>1 April<br>4 April<br>8 April<br>8April<br>10 April<br>12 April<br>17 April<br>2 May<br>5 May<br>6 May
	Women sport	- Women's Sports Podcasts: Throughout this lockdown I have found it hard to find many podcasts that have focused on female sports/athletes, although @FiredUpSport covers many stories from the world of female sport and gives great insight to some interesting characters<br>- I hope the great game i spent a good few years in ans enjoyed helping some great talents gets the assistance it needs grown so much in last few years as has all female sport #GrowTheGame 'FIFPRO: Coronavirus threatens women's football's existence'	15 April<br>16 April

		- "I do think about the young women that had worked all their life to get to this stage." The coronavirus has had a huge impact on women's sport. Germany's Pernille Harder is among those who have described their experiences.<br>- Women's sport must be safeguarded from the economic effects of coronavirus, former Football Association director Dame Heather Rabbatts says.<br>- "Before the coronavirus crisis, we were seeing record levels of activity and the gender gap narrowing. Our most recent figures show more women than men are starting to do less activity."	29 April<br>7 May<br>7 May
	Amateur and community sport	- Non-league Barnet make 60 employees redundant in wake of coronavirus<br>- Sport England are offering support for small (sports focussed) community groups that are affected by Coronavirus<br>- Local #bowls clubs may need volunteers to help with routine maintenance. Locals could rally round. Offer support for mowing and keeping tidy for another day. Many clubs already rely on retired volunteers who may be the 'at risk' category and need to stay safe<br>- NEW: 'Local organisations, like sports clubs, are organised networks of people used to pulling together activities in their community.'<br>- The #coronavirus has pulled down the shutters on events, training and activity across Wales, leaving many clubs and organisations struggling financially. We have some solutions! Here's some info on grants, loans and support to help you.<br>- New funding alert We're making a £20 million Community Emergency Fund available immediately for local club and community organisations. Apply now to bid for grants between £300 and £10,000<br>- @SportScotland have produced this overview of 'Support available to clubs & community sport organisations' It contains links to Scot gov &Uk gov support packages & financial/operational advice & guidance through this period. Check for updates.<br>- The team at Durham Tourism is working to keep the community [and businesses] in #DurhamRegion connected & informed during the COVID-19 pandemic. Our latest enews covers how to #supportlocal and a round up of @sport_durham community initiatives<br>- The types of organisations eligible for the @Sport_England Community Emergency Fund include voluntary and community sector organisations who deliver or enable sport and physical activity. Find out more on our #Covid19 support web page: http://bit.ly/EssexCovid<br>- The Sport Wellbeing Hub is a valuable online resource that we encourage everyone to use across the sporting community, those involved in sport, at all levels and all abilities. Everyone.<br>- Understanding the COVID-19 financial impact on Amateur Sport. This new survey from the Provincial Government seeks specific input from organizations on the economic impacts of the pandemic. Please take the time to share your organization's input.	17 March<br>18 March<br>21 March<br>24 March<br>30 March<br>31 March<br>7 April<br>7 April<br>8 April<br>24 April<br>8 May
	Media	- Anyone else thinking of scaling back @SkyUK subscriptions as you are paying for sport you are not getting? I have decided to continue through @virginmedia but expect my loyalty to be rewarded when things start up again<br>- Nordic pay TV company NENT recently announced it is temporarily reducing the cost of its sports packages due to coronavirus causing postponement of live sports.<br>- With Sky announcing that they will allow customers to pause their sports subscription will Virgin be offering a similar service? With no "Live sport" and many now facing life with no income due to COVID-19 this would be a very welcome gesture!!<br>- In the first of our new 'In Lockdown' series, our team looks at sport - a sector massively impacted by Covid-19 - to examine if there is still an appetite for sports content, and how can brands get to grips with the challenges they're currently facing.<br>- beIN SPORTS has decided to withhold Ligue 1 TV money during the shutdown	14 March<br>17 March<br>20 March<br>26 March<br>4 April

		- There might be profound effect in keeping an entertainment businesses running through these times. Increase the morale of viewers, take their mind off the negativity and help the economy. Who knows if that might save more lives in the long run?...I don't. No one does....	3 May
Health impact	Minimizing effects	- So if schools are to remain open, why the restriction on sport? Let them play #coronavirus<br>- I hate the Coronavirus, not for the fact that it can kill me, but for the fact that it kills weaker people, crashes…<br>- Coronavirus is just like your missus, doesn't let you watch sport or go to the pub, just have to sit at home and do nothing<br>- Heard some really ridiculous stuff since the start of the year. I mean like outrageously ridiculous. Like I heard t…	17 March<br>9 March<br>17 March<br>8 May
	Celebrities supporting anti-Covid campaigns	-Blood Red: 'Look out for each other' - Jurgen Klopp's message to Liverpool fans<br>- Austrian soccer players telling their fans to stay home and stay safe<br>- McCarthy urges public to listen to coronavirus advice as UK goes into lockdown<br>- "I feel like I'm a nice guy and I try and make a joke about social distancing but it's getting to the point where I'm going to have to tell people to go away. This is a very serious matter." @RealCFrampton<br>- Ipswich Town midfielder Emyr Huws has urged everyone to support the 'stay at home, protect the NHS, save lives' campaign as the UK combats the coronavirus outbreak.<br>- "I'm praying for every single one of you guys. Keep everyone in my thoughts and my prayers, and life may keep throwing punches at me but I'm going to keep getting back up." @KarlTowns mom has COVID-19. He wants others to know how serious the illness is.<br>- Newcastle United legend Alan Shearer has echoed the advice of the government and health experts by urging everyone to stay home to combat the coronavirus pandemic.<br>- This virus is affecting so many. Stay home, save lives. [Anthony Joshua]<br>- Tottenham striker Harry Kane urges people to 'stay calm' during coronavirus lockdown	14 March<br>20 March<br>23 March<br>24 March<br>24 March<br>25 March<br>29 March<br>31 March<br>7 April
	Safe and secure practice	- Buy WEST BIKING KN95 Antiviral Coronavirus Sport Face Mask With Filter Activated Carbon PM 2.5 Anti-Pollution Running Cycling Mask<br>- CoolChange Cycling Face Mask KN95 Coronavirus Anti-pollution Sport Training Bicycle Bike Dust Mask Activated Carbon…<br>- The nature of basketball as a sport does not allow for social distancing which is very important to doing our part…<br>- 'I just want people to understand. Stop being childish. Just stay home or keep apart. I've seen what this can do.'…	2 March<br>13 March<br>27 March<br>26 April 1,2551E+18 0,48903[illegible]
	Renewal	- I've always wondered about people who don't watch sport and what they do with their lives. Well in the last couple of days I've realised that they probably just sit around doing nothing all day<br>- Those who have much have much responsibility & opportunity at times like this. Generosity & kindness like this is…<br>- Sport has a duty of care to the UK. Sport must not start up again, to make selfish Sportsmen/women, happy & Richer…<br>- We really need to stop with the sporting euphemisms for covid19 . In the last 5 mins I've heard/ seen "hospital b…<br>"Sport is a big part of our lives, but it's really not that important in comparison to keeping people healthy."<br>- Sport has found a new home... your home. #isolation #isolationsports #COVID19 #coronavirus #sport #worldsport…	14-Mar<br>18 March<br>2 April<br>12 April<br>14 April<br>30 April
		- For now, it doesn't mean you can't still run activities where people gather, and it definitely doesn't mean that people should be	

	Overcoming lockdown	discouraged from being active. #CoronaVirusUpdate from @Sport_England	12 March
		- Great ways to stay active during this time of uncertainty.	17 March
		- Don't let #coronavirus be the thing that stops you getting active, make it the thing that STARTS you getting active. First run in four years, first exercise since no.2 was born in September. Follow @Sport_England and #StayInWorkOut for help, support and guidance.	19 March
		- Looking to keep active at the moment, then as mentioned by the @BBC tennis is a good sport for you	19 March
		- Here's some good advice from @Sport_England on staying active during #coronavirus. Stay active. Stay healthy. Be best prepared for when training for @WeAreInvictus Team UK Training starts again.	19 March
		- Even if you cannot go to Gym, you still got to do exercise. Coronavirus is not an excuse to stop training.	22 March
		- One run can change your day, many runs can change your life. 21 days #StayHomeIndia. Let us just try & do 10min x 2 times home exercise. @olympicchannel Olympic athletes show us how. Really loved this link. Stay positive, Stay fit & Build immunity.	25 March
		- We know people are having to find very different ways to keep active at the moment. Get involved in @Sport_England's new 'Join The Movement' campaign and share how you #StayInWorkOut. Learn more, here: http://orlo.uk/brBLi	26 March
		- Here is my #PressUpChallenge! remember to stay active during the #coronavirus guys, go for a walk or do some home workouts!	26 March
		- Laois Sports Partnership is looking for new ways to keep people healthy and active at home. Got an idea or challenge? See full post for full competition details with sports equipment vouchers up for grabs #UnitedInSport #BeActive #Sponsored	27 March
		- People can find more ways to get active around their home thanks to @Sport_England's new 'Join The Movement' campaign	28 March
		- It's important to stay active during #COVID19, if you're going out to stay active, do so only once a day and stay 2 metres apart from anyone. If you can't get out, @Sport_England have some great ways to stay healthy at home.	30 March
		- As people are asked to stay home to prevent the spread of #COVID19, many are looking for new ways to stay physically active. Ahead of the Int'l Sports Day for Development & Peace, see how to #BeActive amid the #coronavirus pandemic	3 April
		- I think there's a lot of sense in this. We must keep exercising to stay fit and healthy.	8 April
		- 63% of adults in England say it's more important to be active now, compared to before #coronavirus.	15 April
		- It's important to stay active during #COVID19, if you're going out, do so only once a day and stay 2m apart from anyone not in your house. If you can't get out, @Sport_England and @ActiveWCC have some great ways to stay #HealthyAtHome	21 April
		- Now, more than ever, it's important to have work/life balance – which can be challenging in the best of times, let alone when work & life are happening within the same 4 walls. Our staff are enjoying themselves via sport, nature, art & more!	7 May
