# Supplementary figures and images for: The Impact of COVID-19 on Sport in Twitter: A Quantitative and Qualitative Content Analysis
Source: Int J Environ Res Public Health. 2021 Apr 25;18(9):4554. doi: 10.3390/ijerph18094554 (PMC8123335; doi:10.3390/ijerph18094554)

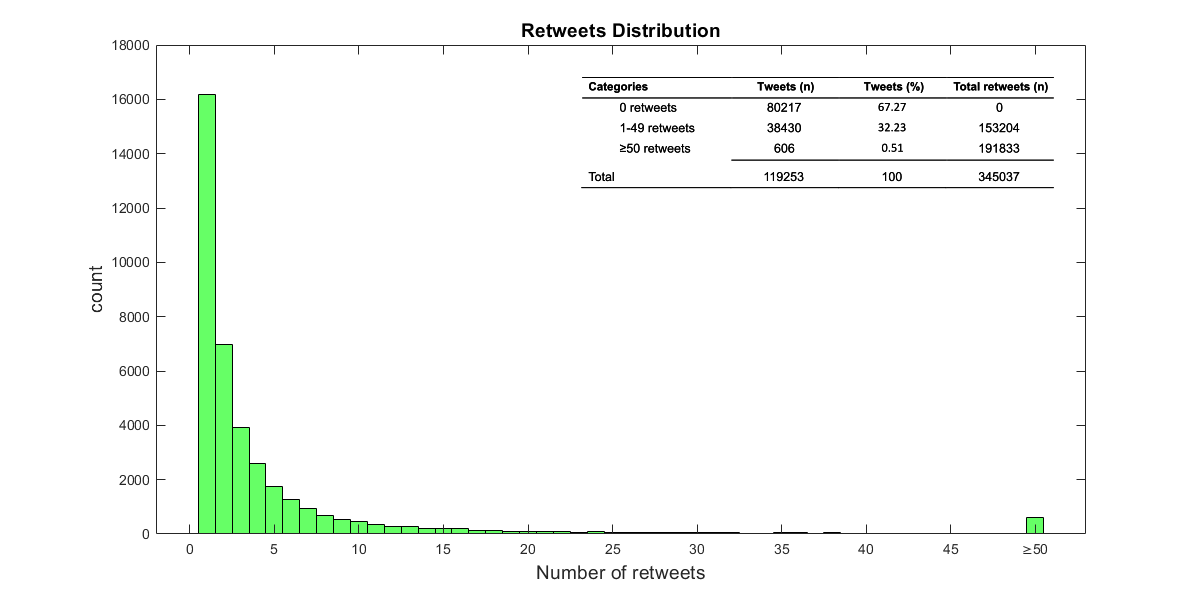

Supplement: Supplementary file 1 [file ijerph-18-04554-s001.zip › Figure S1_retweets_distribution.png]
